# Rationally minimizing natural product libraries using mass spectrometry

**DOI:** 10.1128/msystems.00844-24

**Published:** 2025-02-24

**Authors:** Monica Ness, Thilini Peramuna, Karen L. Wendt, Jennifer E. Collins, Jarrod B. King, Raphaella Paes, Natalia Mojica Santos, Crystal Okeke, Cameron R. Miller, Debopam Chakrabarti, Robert H. Cichewicz, Laura-Isobel McCall

**Affiliations:** 1 Department of Chemistry and Biochemistry, University of Oklahoma, Norman, Oklahoma, USA; 2 Department of Chemistry and Biochemistry, San Diego State University, San Diego, California, USA; 3 Burnett School of Biomedical Sciences, University of Central Florida, Orlando, Florida, USA; 4 College of Pharmacy, University of Michigan, Ann Arbor, Michigan, USA; University of California San Diego, La Jolla, California, USA

**Keywords:** metabolomics, fungi, drug discovery, specialized metabolites, mass spectrometry, computational techniques

## Abstract

Natural products are a critical source of novel chemotypes for drug discovery. However, the implementation of natural product extract libraries in high throughput screening is hampered by natural product structural redundancy and potential for bioactive re-discovery. This challenge and large library sizes drastically increase the time and cost during initial high throughput screens. To address these limitations, we developed a method that leverages liquid chromatography-tandem mass spectrometry spectral similarity to dramatically reduce natural product library size, with minimal bioactive loss, and applied this to a collection of fungal extracts. Importantly, this method also afforded increased bioassay hit rates against microbial targets, with broad applicability across assays and natural product sources. Thus, this method offers a broadly applicable strategy for accelerated and cost-effective natural product drug discovery.

**IMPORTANCE:**

Natural product libraries are large collections of extracts derived from fungi, plants, bacteria, or any other natural sources. These libraries play an important role in the initial phases of drug discovery, providing the basis for bioassays against a target of interest. However, these collections often comprise thousands of extracts with sometimes overlapping chemical structures, which can result in a bottleneck in both time and costs for the initial phases of drug discovery. Here, we have developed a method that uses mass spectrometry to dramatically reduce the size of these libraries, with minimal tradeoffs and improved success rates in bioassays. Ultimately, this will speed up the process of bioactive candidate identification and isolation, and drug development overall.

## INTRODUCTION

Natural products play a major role in the development of novel pharmaceutical agents, accounting for nearly 70% of newly approved drugs in the past 40 years as direct natural molecules or as natural product mimics (1). Natural product discovery pipelines typically begin by screening large libraries of extracts (crude or pre-fractionated), then identifying and isolating bioactive candidates from these extracts. However, large libraries can result in long development times, high costs, and duplicate drug candidate identification (rediscovery). Prior efforts to address these challenges often focused on the initial collection of library extracts, rather than on rationally reducing library size. These prior studies analyzed the impact of factors like geography, phylogenetics, or culturing conditions on small molecule diversity, using approaches like molecular networking, principal coordinate analysis, principal component analysis, and hierarchical clustering, for example, to select optimal culture conditions (e.g., references 2–4). Considerable efforts have also focused on DNA-based approaches to prioritize or select samples, for example, relying on biosynthetic gene clusters (e.g., reference 5). Clark et al. and Costa et al. combined DNA sequencing with Matrix-assisted laser desorption ionization–time of flight-based protein analysis to reduce redundancy (6–8). However, none of this prior work evaluated their methods in terms of applicability of the reduced libraries in the context of high-throughput screening hit rates or retention of potential bioactive candidates and often required complex pipelines with multiple forms of data collection.

Instead, we sought to develop a method that only requires MS/MS spectral data to design natural product extract libraries and tested its applicability in real-world drug development activity screening assays. Here, we report the development of a new method to rationally reduce the size of natural product extract screening libraries by directly addressing cross-organismal redundancy in small molecule natural product production, through liquid chromatography-tandem mass spectrometry (LC-MS/MS) and molecular networking (9). This method led to (i) little loss of diversity, (ii) little loss of bioactive candidate molecules, (iii) increased bioactivity hit rate for a variety of whole-organism and purified protein targets, and (iv) greater library size reduction than previously published methods (6, 10).

## RESULTS AND DISCUSSION

**Fig 1:**
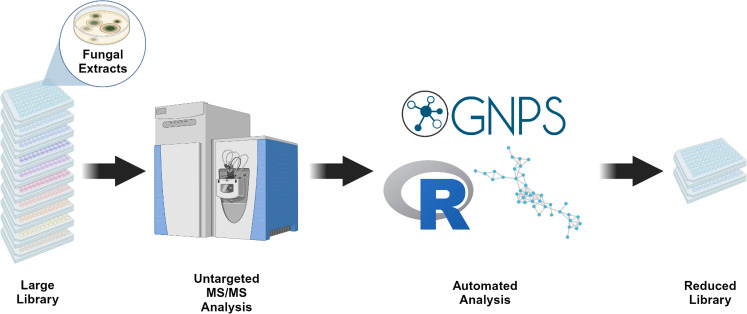
Method conceptual overview.

Our method uses an untargeted LC-MS/MS approach to choose extracts from a large natural product extract library. These extracts are complex and contain large numbers of different small molecules, some of which may be bioactive. Starting with MS/MS fragmentation patterns, data are then processed through GNPS classical molecular networking software to group MS/MS spectra into scaffolds (Fig. 1). These scaffolds are based on MS/MS fragmentation similarity, which correlates to structural similarity (9). Unlike approaches by Anderson et al. or Ito et al. (11, 12), our rational libraries focus on scaffold diversity, as molecules with similar structures often demonstrate similar biological activity (13–15). Adducts and in-source fragments of the same molecule with similar MS/MS fragmentation will group together in the same scaffold in this approach, leading to less duplication in the rational library than if this method was applied to individual molecular signals. Additionally, many drug development pipelines synthetically modify natural product scaffolds to improve structure-activity relationships (16). Therefore, diversifying core scaffolds, rather than individual molecules, can be prioritized in the initial library design. Our method also differs from prior work maximizing the diversity of known chemical structures in libraries (17–19), since our method does not require a priori structure elucidation.

**Fig 2:**
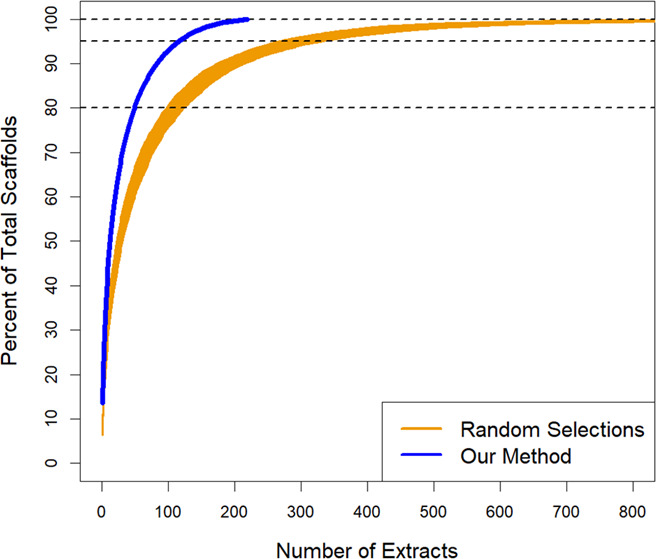
Rapid aggregation of scaffold diversity using our method, outperforming random selection.

Using custom R code, which we make freely available (see Data Availability), our method selects the natural product extract with the greatest scaffold diversity. Next, the extract that contains the most scaffolds not already accounted for is added to the rational library. This process is automatically iterated until a desired percentage of scaffold diversity is reached in the rational library or maximal scaffold diversity is achieved. Compared to random selection, this accelerates the accumulation of diversity and offers an 84.9% reduction in the library size needed to reach maximal scaffold diversity. Specifically, in our evaluation library of 1,439 fungal extracts, random sample selection achieved 80% of maximal scaffold diversity with an average of 109 extracts, whereas our method reached the same level of diversity with only 50 extracts. Similarly, to reach 100% scaffold diversity (representation in the non-reduced library of all detected scaffolds), random selection required an average of 755 extracts. Our method only requires 216 extracts (Fig. 2; Table S1). Thus, a rational library of 216 extracts would achieve the same scaffold diversity as the full library of 1,439 extracts, a 6.6-fold reduction in size. In a scenario where 80% diversity is acceptable, this would represent a 28.8-fold library size reduction, from 1,439 extracts to 50 extracts. This size reduction far exceeds the size reduction achievable with alternative methods (6)

Such dramatic library size reduction leads to concerns that key bioactive extracts will be lost. To assess bioactive extract loss in our rational libraries, we compared the bioactivity hit rate of the full library and of our minimal rationally designed library on the eukaryotic parasites *Plasmodium falciparum* and *Trichomonas vaginalis*, as well as the influenza virus enzyme neuraminidase. Importantly, these assays represent two of the major types of assays in high throughput screening: phenotypic assays (*P. falciparum* and *T. vaginalis*), and target-based assays on purified enzymes (neuraminidase). To prevent bias, rational minimal library selection was blinded to bioactivity scores.

In the full library, the hit rate against *P. falciparum* was 11.3%, whereas the rational library designed to capture 80% scaffold diversity had an increased hit rate of 22%, and the rational library to 100% diversity had a hit rate of 15.7%. This pattern was replicated against *T. vaginalis* and neuraminidase (Table 1): our method increased the hit rate from 7.64% for the full library against *T. vaginalis*, to 18% in the 80% scaffold diversity library. A similar increase from 2.57% to 8% was observed for the full library versus the 80% scaffold diversity library against influenza virus neuraminidase, confirming that the increased hit rate is observed across a range of screening campaign designs and baseline hit rates in the full library (Table 1). Therefore, our method chooses extracts more likely to contain bioactivity, potentially because our method reduces the chemical redundancy inherent in natural product libraries (2, 6). The higher hit rates in the 80% maximum diversity library could also be a result of the algorithm adding the most diverse extracts to the rational libraries first, whereas the later additions contain less diversity, but more rare scaffolds. These scaffolds may not be active in the assay tested but could be active in other assays. To confirm that these findings are not merely an artifact of the smaller library size, we compared our method with 1,000 iterations selecting the same number of random extracts. In all cases, our method outperformed random extract selection (Table 1; Table S2).

**TABLE 1:** Higher hit rates for rational libraries

Activity assay	Hit rate in full library (1,439 extracts)	Hit rate in the 80% scaffold diversity library (50 extracts)	Lower and upper quartile hit rates for 50 random extracts (1,000 iterations)	Hit rate in the 95% scaffold diversity library (116 extracts)	Hit rate in the 100% scaffold diversity library (216 extracts)
*P. falciparum*	11.26%	22.00%	8.00–14.00%	19.83%	15.74%
*T. vaginalis*	7.64%	18.00%	4.00–10.00%	17.24%	12.50%
Neuraminidase	2.57%	8.00%	0.00–2.00%	6.03%	5.09%

To further address concerns with regards to loss of bioactive molecules, we identified features correlated with bioactivity in the full library, and assessed whether they were retained in the minimal rational library. In this case, “feature” is defined as a unique *m/z* and retention time combination found in the MS data (see “Bioactivity correlations”). We found that, of the 10 features significantly correlated with anti-*Plasmodium* activity in the full library (ρ > 0.5, *P* < 0.05, FDR corrected), 8 were retained in the 80% scaffold diversity library, and all were retained in the 95% and 100% scaffold diversity libraries. A similar conclusion was reached with regard to molecules correlated with anti-*T*. *vaginalis* or anti-neuraminidase activity (Table 2). Using this information, we were also able to identify and dereplicate known active molecules, using methods previously described (20, 21) (Fig. S1). No overlap was observed between features significantly correlated to bioactivity in each assay (Fig. S1).

**TABLE 2:** High retention of bioactive candidate molecules in our rational libraries, for three different activity assays

Activity assay	Features found to be significantly correlated to activity in full data set	Retained in the 80% scaffold diversity library	Retained in the 95% scaffold diversity library	Retained in the 100% scaffold diversity library
*P. falciparum*	10	8	10	10
*T. vaginalis*	5	5	5	5
Neuraminidase	17	16	16	17

**Fig 3:**
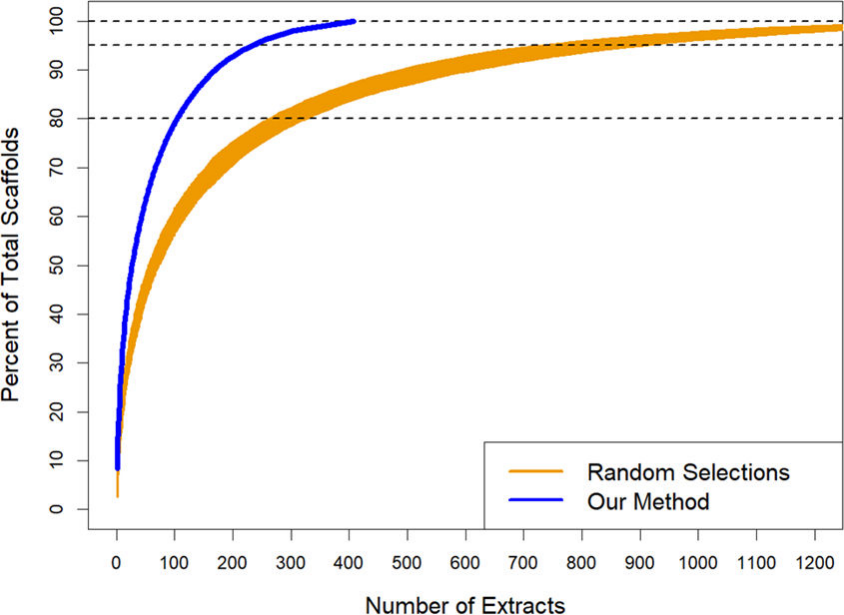
Rapid accumulation of scaffold diversity with our rational library building method, applied to publicly available data.

To validate our method and demonstrate its utility beyond our laboratory, we applied the same rational library-building process to LC-MS data collected by independent investigators and tested for bioactivity against another pathogen, the parasite *Trypanosoma cruzi* (22). Notably, their library consisted of pre-fractionated plant samples, rather than crude fungal samples. After rationally reducing their full library size (1,600 total extracts) down to 104 extracts with our method (Fig. 3; Table S3) to achieve 80% maximal scaffold diversity, we also observed a rapid accumulation of chemical diversity, an increased hit rate in the rational libraries when compared to the full library (from 0.5% to 1.92%), and the retention of 90–100% of the features correlated with bioactivity (Tables 3 and 4). These results confirm the utility of our method across pathogens and natural product sources and also in assays with lower initial hit rates.

**TABLE 3:** Higher hit rates in rational libraries, applied to publicly available data

Activity assay	Hit rate in full library	Hit rate in the 80% scaffold diversity library	Hit rate in the 95% scaffold diversity library	Hit rate in the 100% scaffold diversity library
*T. cruzi*	0.5%	1.92%	1.72%	1.47%

**TABLE 4:** High retention of bioactive candidate molecules in rational libraries, applied to publicly available MS/MS data with *T. cruzi* activity data

Activity assay	Features found to be significantly correlated to activity in full data set	Retained in the 80% scaffold diversity library	Retained in the 95% scaffold diversity library	Retained in the 100% scaffold diversity library
*T. cruzi*	21	19	21	21

In addition to the accelerated screening enabled by our rational library development method, there are also many other advantages of the LC-MS/MS data acquired through this pipeline. Any bioactive candidates identified in a screen of these rational libraries will already have LC-MS/MS data readily available for initial structure elucidation. This data can also be input into many convenient platforms for dereplication (23, 24) and structural annotation (9, 25). For example, we readily identified leucinostatin- and altersetin-related molecules in this data, which have known antimicrobial activity (26, 27) (Fig. S1). If their rediscovery is undesirable, the extracts containing these scaffolds could be excluded from the rational library. Alternatively, in under-studied diseases, investigators may wish to retain these extracts, to enable repurposing of these known compounds to other disease models. Likewise, extracts containing molecules previously described as promiscuously toxic in the target biological system can also be excluded, to prevent rediscovery. Additionally, the MS data can be put into other convenient platforms for adduct and in-source fragment identification, further narrowing features of interest for isolation (28, 29). Abundance information generated from LC-MS data can also be leveraged to build bioactive correlations (21) and identify candidate bioactive molecules, as demonstrated in this study. Such data can be used to guide compound isolation and purification for definitive structure elucidation by NMR.

The rational libraries presented above were built with positive ionization data, as more molecules can be detected in positive mode (30) (Fig. S2). We detected 45,601 features and 5,126 scaffolds in positive mode, whereas negative ionization detected 33,149 features and 3,530 scaffolds. Despite this, we found the same conclusions when analyzing libraries built with negative mode data (Tables S1 and S4; Fig. S3; Data S1). Likewise, modulating GNPS classical molecular networking parameters (9) found similar conclusions even after each parameter adjustment (Data S2). To test if our reduction method would also be functional with low-resolution mass spectrometers, we adjusted the classical molecular networking fragment and precursor *m/z* tolerance parameters to mimic low-resolution data (Table S5). This adjustment had limited impact on rational library hit rate increases and retention of bioactive candidates. This suggests that our minimization method does not require high-resolution MS data acquisition for effective results (Tables S1 and S6; Fig. S4; Data S1). These observations indicate a broad tolerance of our approach to data acquisition and processing parameters.

Certain fungal genera, like *Penicillium*, *Pseudogymnoascus*, and *Trichoderma*, are more likely to be selected for the rational libraries, while others, like *Mucor*, are less likely to be selected for the rational libraries (Data S3). This suggests that with our fungal culturing methods, some genera are less likely to produce unique scaffolds, as expected, and therefore are not prioritized using our selection method. Additionally, certain genera were more likely to be active in our activity assays, beyond their relative presence in the libraries (Fig. S5). Extracts that are not a hit in a given assay are however still valuable, as there was a minimal overlap of active extracts between activity assays, albeit more between *P. falciparum* and *T. vaginalis* (Fig. S6).

Geographically, the distribution of our fungal extracts was very broad, with extracts from almost every state. Active extracts from each assay did not significantly concentrate in one climate or geographic region (Fig. S7 and S8). Many of our active extracts were close to urban areas, notably in the Dallas, Oklahoma City, and Columbus counties. However this is likely due to ease of collection from the Citizen Science participants and increase in samples sent from those in urban areas, rather than innate bioactivity from urban fungi.

**Fig 4:**
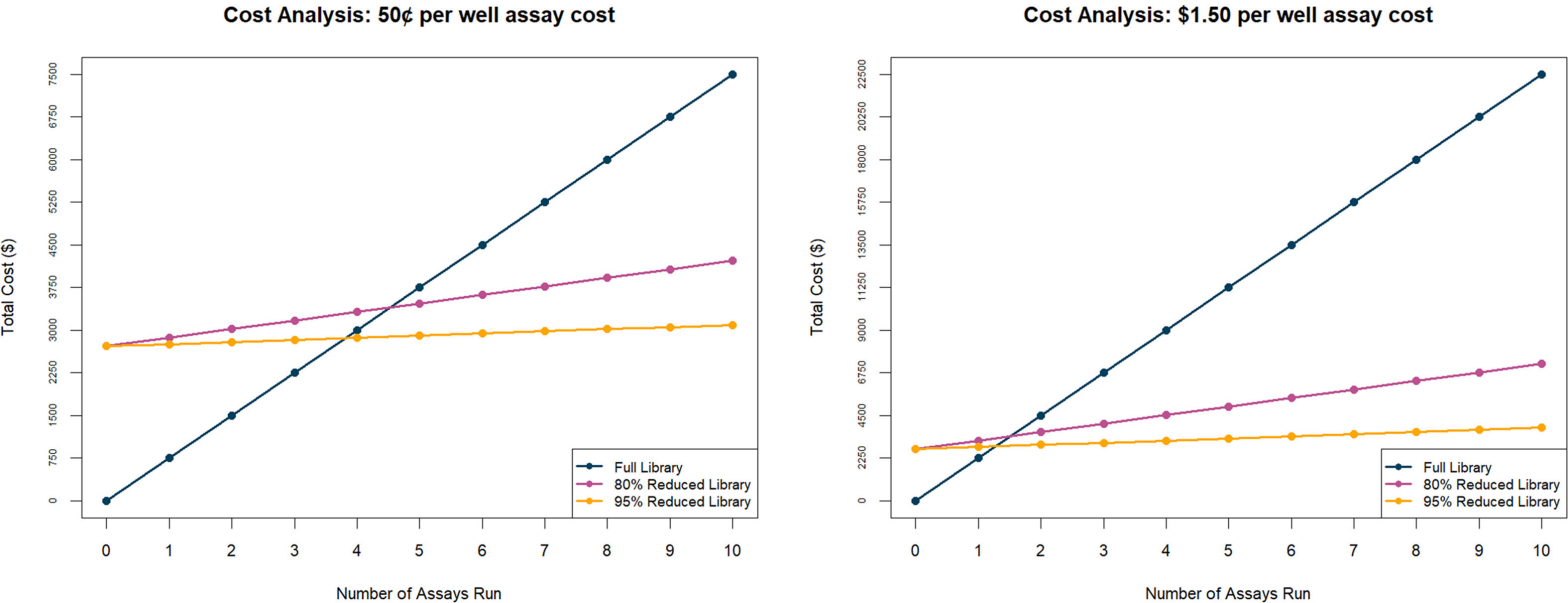
Cost analysis for the rational library approach accrues after a few screening campaigns. This is a cost analysis of our reduction method with a library size of 1,500 extracts. Assuming an 80% or 95% library size reduction can be achieved (see Table 1; Table S3) and a cost-per-well average of $0.50 or $1.50 per assay, the upfront cost of LC-MS analysis can be rapidly justified.

While library screening costs represent a small proportion of the total drug development investment, they remain a preliminary bottleneck that is especially critical in settings where financial resources are limited, such as neglected tropical diseases or rare conditions. We calculated the costs of the supplies used for LC-MS data acquisition on the full library to $1.81 per extract, for an estimated total cost of $2,604.59 to acquire LC-MS data for all 1,439 extracts. While this was more costly than the *T. vaginalis* and *P. falciparum* bioactivity assays (estimated at $0.061 per extract and $0.17 per extract, respectively), the neuraminidase assay was much more costly, at $5.35/extract. Dreiman et al. note an average cost per well of $1.50 for phenotypic assays (31). At such an average assay cost per extract, marginal cost-savings accrue from our approach by two assays, with noticeably greater cost savings within three assays (Fig. 4). At a lower assay cost per well of $0.50, our rational method provides cost savings within four assays. Given the challenges of establishing natural product screening libraries, the anticipation is that they will be used for many different diseases, and thus performing multiple assays with the same screening library is standard procedure. In addition, by reducing the size of the library to be screened using our method, additional screening, dose-response assessment, and counter-screening assays can be implemented, leading to reduced false positive rates. Rationally designed library size reduction also enables the implementation of more complex (and thus more costly per-well) assays that better mimic *in vivo* conditions, increasing translatability and likelihood of clinical success.

Thus, while we acknowledge the initial up-front cost and time needed for the LC-MS analysis, this is mitigated by the fact that it can enable cost reduction across all subsequent high throughput screening projects using this library (as demonstrated here), prevent rediscovery of known bioactive or pan-cytotoxic compounds, and expedite bioactive candidate structure elucidation. Our method should also be suitable for libraries of pre-fractions, or to prioritize crude extracts for fractionation, leading to further cost reduction and time savings. The method can be further expedited by refining the separation method, multiplexing LC columns or introducing advances such as microfluidic separation (32). Finally, our method was applicable to data sets and screening assays implemented outside our laboratory, demonstrating its broad utility.

## MATERIALS AND METHODS

### Collection of fungal extracts

The University of Oklahoma’s Citizen Science Soil Collection Program (33) asked citizen scientists to submit soil samples that were then used to isolate fungi. These fungi were grown to generate extracts that are used in various drug discovery efforts. Fungi were selected for the library based on morphological analysis, and the unique fungi from each soil sample were retained. ITS-based taxonomic identifications were also generated. Over 79,000 fungi originating from all 50 states and the District of Columbia are included in the fungal library (Citizen Science Soil Collection Program [shareok.org]). The subset of fungal extracts used in this study was isolated from 443 soil samples with a broad distribution across the United States (Fig. S7) and includes members of 141 different genera (Data S4). Taxonomic identification was accomplished via sequencing of the internal transcribed spacer region of the genomic DNA (11). These fungal isolates were cultured for 3 weeks on a solid-state medium composed of Cheerios breakfast cereal supplemented with a 0.3% sucrose solution containing 0.005% chloramphenicol, as previously optimized (34, 35).

### Metabolite sample preparation

Samples for bioactivity assays and LC-MS analysis were prepared on an automated platform that combined both extraction and partitioning steps as previously described (4). Fungal cultures prepared in 16-by-100 mm borosilicate tubes were subjected two times to a vol:vol water:ethyl acetate partitioning. The aqueous portion was discarded. The organic solvent was removed *in vacuo* and the remaining organic residues were stored at −20°C until resuspended in DMSO for screening. These same extracts were diluted 1:10 in methanol containing 2 µM sulfadimethoxine as an internal standard for LC-MS/MS analysis.

### *P. falciparum* assay

Parasites were cultured following a protocol by Trager and Jensen, with minor modifications (36, 37). Specifically, the multidrug-resistant *P. falciparum* line Dd2 was grown at 37°C in 5% CO_2_ with Rosewell Park Memorial Institute 1640 media supplemented with 25 mM of 4-(2-hydroxyethyl)-1-piperazineethanesulfonic acid (pH 7.4), 26 mM NaHCO_3_, 2% dextrose, 15 mg/L hypoxanthine, 25 mg/L gentamicin, and 0.5% Albumax II in human A+ blood. The impact of fungal extracts on *P. falciparum* growth was measured via a SYBR Green I fluorescence-based assay as described (36). Extracts were resuspended in DMSO and plated on microtiter plates with asynchronous Dd2 culture at 1% parasitemia, 1% hematocrit, for a final concentration of 2 µg/mL, maintaining a DMSO concentration of <0.25% in all cases to avoid assay interference. Culture plates were then incubated with extracts under standard growth conditions for 72 h. After incubation, assay plates were frozen at −80°C and subsequently thawed to promote lysis. Once thawed, an equal volume of lysis buffer (20 mM Tris-HCl, 0.08% saponin, 5 mM ethylenediaminetetraacetic acid, and 0.8% Triton X-100) with 1× SYBR Green I was added. Plates were incubated for 45 min to 1 h, protected from light, prior to fluorescence reading at excitation 485 nm, emission 530 nm, on a Synergy Neo2 multi-mode reader (BioTek, Winooski, VT, USA). Values were then normalized to 10 mM chloroquine (positive control) and vehicle-only (negative control) wells. Extracts showing more than 75% inhibition of parasite growth were considered hits. Bioactivity assays were performed separately from rational library generation; investigators performing this assay were unaware of the rationally selected extracts, and vice versa.

### *T. vaginalis* assay

*T. vaginalis* Donne (PRA-98) from the American Type Culture Collection (ATCC, Bethesda, MD, USA) was grown at 37°C in filter-sterilized Keister’s modified TYI-S33 medium (2% [wt/vol] casein, 1% [wt/vol] yeast extract, 55.6 mM glucose, 34.2 mM NaCl, 4.4 mM KH_2_PO_4_, 5.7 mM K_2_HPO_4_, 12.7 mM l-cysteine HCl, 1.1 mM l-ascorbic acid, 86.7 µM ferric ammonium citrate, 10% [vol/vol] heat-inactivated fetal bovine serum, 0.052% [wt/vol] bovine bile salts in 1 L of Millipore water). Micro aerophilic conditions were maintained with the use of BD GasPak EZ Campy sachets. Samples consisting of 4 × 104 trichomonads per well were treated with DMSO, 25 µM metronidazole, or fungal extracts at 10 µg/mL, not exceeding 0.5% DMSO. After a 16-h incubation, the cells were fixed (1% glutaraldehyde, 5 µM propidium iodide, and 5 µM acridine orange in phosphate-buffered saline). After a 3-h incubation at 37°C, the cells were imaged using a PerkinElmer Operetta high-content imaging system. Data were analyzed using the Harmony 3.5.1 software package as previously described (35). The number of live cells imaged was normalized to the DMSO control (100% growth). Extracts were considered active if they inhibited the growth of *T. vaginalis* by more than 80%. Bioactivity assay was performed separately from rational library generation; investigators performing this assay were unaware of the rationally selected extracts, and vice versa.

### Neuraminidase assay

Fungal extracts plus 0.01% TritonX to break up aggregates were tested in high throughput format at 10 µg/mL using a commercial neuraminidase activity assay, according to the manufacturer’s instructions (Sigma Aldrich, catalog number MAK121). Those identified as reducing neuraminidase activity to less than 30% of vehicle control were retested in a dose-response curve to verify activity. Hits were considered confirmed if they showed dose-dependent inhibition. Briefly, the reaction mixture containing buffer, substrate, cofactors, enzyme, and dye was mixed with the extracts and incubated at 37°C. Absorbance at 570 nm was recorded at 20 and 50 min. The activity of the neuraminidase enzyme was calculated according to the kit instructions. Activity scores were then normalized such that the positive and negative controls were set to 0% and 100%, respectively. Bioactivity assay was performed separately from rational library generation; investigators performing this assay were unaware of the rationally selected extracts, and vice versa.

### LC-MS/MS data acquisition

Fungal extract separation was done with Thermo Scientific Vanquish ultra-high-performance liquid chromatography instrument equipped with a Kinetex 1.7  µm C18 50 × 2.1 mm LC column with a C18 guard cartridge (Phenomenex). The mobile phases used were water with 0.1% formic acid (mobile phase A), and acetonitrile with 0.1% formic acid (mobile phase B). The following 12.5-min gradient was used: (i) 0–1 min, 5% B; (ii) 1–8 min, linear increase to 100% B; (iii) 8–10 min, 100% B; (iv) 10–10.5 min, linear decrease to 5% B; and (v) 10.5–12 min, 5% B. A flow rate of 0.5 mL/min was maintained, and the column chamber temperature was kept at 40°C.

For MS/MS acquisition, a Q-Exactive Plus (Thermo Scientific) high-resolution mass spectrometer was used. Both positive and negative ionization data were collected, with the parameters shown in (Table S7). Data acquisition was performed in randomized order. Injection volume was 5 µL. A blank and pooled quality control samples were analyzed every 12 injections to monitor instrument performance. Retention time shifts were analyzed by using a mix of sulfadimethoxine, sulfachloropyridazine, sulfamethazine, sulfamethizole, amitriptyline, and coumarin-314 standards. This standard mix was taken at the beginning of the run, every 100 samples throughout the run, and at the end of the run.

The Q-Exactive Plus was calibrated using Pierce LTQ Velos ESI positive and negative ion calibration solution (ThermoFisher) prior to analysis.

### Data processing

Raw files were converted to mzML files using MSConvert (38). Classical molecular networking was performed using GNPS (9). The parameters used are listed in Table S5, and we found that varying the parameters did not significantly impact rational library building (Data S2). Molecular networks were visualized using Cytoscape version 3.9.1 (39) . All data processing was done in Rstudio version 4.3.0 or Jupyter Notebook 6.5.4. The map of soil sample collection and active extract locations was done in qGIS 3.30.3.

Rational Libraries were generated using an R studio code made freely available (see Data Availability). This code uses the node table from the classical molecular networking job, which summarizes each feature, its scaffold family, and which extracts contain the feature. Instructions for downloading the node table are in Fig. S9. To choose extracts to be added to the rational library, the code aggregates the features by scaffold, then chooses the extract that contains the most scaffolds. Then, those scaffolds are deleted from the data set. After that, the extract with the most scaffolds not already accounted for is added to the rational library. This process repeats until a desired percent of maximum diversity is reached or maximum diversity.

Figure 1 was generated using BioRender.com.

### Bioactivity correlations

The quantitative feature data were generated using MZmine 2.53 (40) using the parameters listed in Table S8. The positive and negative ionization data were processed separately. To generate the quantitative table, highly repetitive *m/z*s with close retention times, indicating background or oversplit features, were removed. A fivefold blank removal was applied, removing peaks that can be attributed to the solvent and cheerio media. All data were normalized to the total signal in each extract (TIC normalization).

It is possible that the same molecule can produce multiple features, in the form of different adducts and/or in-source fragments (ISFs). This represents a limitation in our bioactivity correlation and retention analysis. However, if choosing features for isolation, this issue can be identified, because different adducts and ISFs often appear in the same scaffold due to similar MS/MS fragmentation.

Bioactivity correlations were done in R studio with code that is also freely available at https://github.com/mmness/RationalLibraryBuilding, and were loosely adapted from the method described by Nothias et al. (21). In their studies, bioactivity scores of extract features were calculated and imported into a feature-based molecular network (41)to identify bioactive scaffolds. Our method is similar in using quantitative tables generated from MZmine to predict bioactive features. However, there are some key differences. First, we filtered out features present in less than three extracts. While these may represent bioactive candidates, an accurate correlation coefficient cannot be calculated with less than three points. Second, we applied a Spearman correlation instead of a Pearson correlation. Unlike Pearson correlations, which assume linear relationships and normally distributed data, metabolomics data often exhibits logarithmic or exponential relationships (42). Therefore, Spearman correlations are more suitable for our calculations. Finally, we analyze the retention of the bioactive molecules in the rational libraries, which was not performed by Nothias et al. (21).

The code requires the input of the activity data for one of the assays. Then, it builds a Spearman correlation between the number of feature signals and the activity. It also calculates and corrects the *P* value of each feature to activity. The features are filtered based on an FDR-adjusted *P* value less than 0.05, and a Spearman correlation statistic greater than 0.5 (positively correlated with activity).

Bioactivity scores were calculated for both positive and negative ionization MS data, and the data represented in Table 2 show the sum of significant features from both ionization methods. For information about whether the feature was detected in positive or negative mode, see Data S1.

For the publicly available data analysis, the MZmine quantitative table provided by the authors was used (22). Rather than a Spearman correlation, a Pearson’s Bivariate correlation coefficient was calculated. This is because the *T. cruzi* activity data provided was presented as binary data rather than continuous. The data presented in Table 4 represent only positive ionization data, as negative data were not collected on these samples.

The codes to process the MZmine quantitative table, and bioactivity correlation calculations are available at https://github.com/mmness/RationalLibraryBuilding.

## Data Availability

The .raw and .mzML data files used in this article are available in the MassIVE repository under accession numbers MSV000091950 (positive data) and MSV000091980 (negative data) (massive.ucsd.edu). The classical molecular networking jobs are available at https://gnps.ucsd.edu/ProteoSAFe/status.jsp?task=37a75c380d7c464ba278433c6434f7c1 (fungal extracts, positive data, and parameters listed in Table S5), https://gnps.ucsd.edu/ProteoSAFe/status.jsp?task=ab2cceee30e347d185e17d3e2dc95092 (fungal extracts, negative data, and parameters listed in Table S5), and https://gnps.ucsd.edu/ProteoSAFe/status.jsp?task=8ad5a0af7cbc4859a7b943fcaebb7e9d (low-resolution mimicking data, fungal extracts, positive data, and parameters listed in Table S5). The jobs used to verify parameter sensitivity are listed in Data S2. The publicly available data (22) used to verify our method is available in the MassIVE repository under accession number MSV000087728. The classical molecular networking job is available at https://gnps.ucsd.edu/ProteoSAFe/status.jsp?task=a0349e711a214913b0222041e76c2346. The codes used, including for library building and bioactivity correlations, are available at https://github.com/mmness/RationalLibraryBuilding. The bioactivity correlation code is loosely based on the technique presented by Nothias et al. (21) (see "Bioactivity correlations"). The GenBank accession numbers used for sequencing are PP664564–PP665462.

## SUPPLEMENTAL MATERIAL

Supplemental Data Sheets msystems.00844-24-s0001.xlsx Data S1 to S4.

Supplemental material msystems.00844-24-s0002.pdf Tables S1 to S8, Fig. S1 to S9, and captions for Data S1 to S4.

ASM does not own the copyrights to Supplemental Material that may be linked to, or accessed through, an article. The authors have granted ASM a non-exclusive, world-wide license to publish the Supplemental Material files. Please contact the corresponding author directly for reuse.
