# Supplementary material for: Rationally minimizing natural product libraries using mass spectrometry
Source: mSystems. 2025 Feb 24;10(3):e00844-24. doi: 10.1128/msystems.00844-24 (PMC11915828; doi:10.1128/msystems.00844-24)
Supplement: Supplemental material — Tables S1 to S8, Fig. S1 to S9, and captions for Data S1 to S4. [file msystems.00844-24-s0002.pdf]

Supplementary Information for Rationally Minimizing Natural Product Libraries Using Mass Spectrometry

This pdf includes:

<u>Supplemental Tables 1-8</u>

Table S1: Summary of rational library sizes with different parameters for scaffold building.

Table S2: Hit rates for random sampling of positive mode fungal data.

Table S3: Summary of rational library sizes using publicly available data.

Table S4: Higher hit rates for rational libraries, for negative mode analysis of fungal samples.

Table S5: Classical molecular networking parameters.

Table S6: Higher hit rates for rational libraries, using low-resolution mimicking parameters and positive mode analysis of fungal samples.

Table S7: Q-Exactive plus parameters used for fungal data collection.

Table S8: MZmine 2.53 parameters used for feature identification

<u>Supplemental Figures 1-9</u>

Fig. S1: Number of features significantly correlated to bioactivity from each assay, along with known molecule derivatives identified in the analysis

Fig. S2: Annotation overlap between positive and negative ionization modes.

Fig. S3: Rapid accumulation of scaffold diversity with our rational library building method, for negative mode analysis of fungal samples.

Fig. S4: Rapid accumulation of scaffold diversity with our rational library building method, using low-resolution mimicking parameters and positive mode analysis of fungal samples.

Fig. S5: Comparison of fungal genera presence in bioactive samples versus the full library.

Fig. S6: Venn diagram showing active extracts and their overlap between assays

Fig. S7: Fungal samples collection sites.

Fig. S8: Fungal samples collection sites, for active extracts

Fig. S9: Instructions for node table download from the classical molecular networking job page

In excel workbook:

<u>Supplementary Data 1-4</u>

Supplemental Data Sheet 1: Significant bioactive features for each ionization method.

Supplemental Data Sheet 2: Changing classical molecular networking parameters has little impact on library size and hit rates.

Supplemental Data Sheet 3: Genera origins of full and rational libraries: Summary of each genera count/percentages in the full libraries, and the count/percentages in the rational libraries.

Supplemental Data Sheet 4: Fungal metadata.

Supplemental Tables

Table S1: Summary of rational library sizes with different parameters for scaffold building. Both positive and negative mode data were collected. Applying our method to the negative mode data, and to low resolution-mimicking data (based on data processing parameters) resulted in similar reduction effectiveness with regards to library size reduction.

	Number of Extracts to 80% Maximum Scaffold diversity	Number of Extracts to 95% Maximum Scaffold diversity	Number of Extracts to 100% Maximum Scaffold diversity
Positive Mode Fungal Data	50<br>(96.5% reduction)	116<br>(91.9% reduction)	216<br>(85.0% reduction)
Negative Mode Fungal Data	41<br>(97.1% reduction)	96<br>(93.3% reduction)	174<br>(87.9% reduction)
Positive Mode Fungal Data: Low Resolution-Mimicking Data	44<br>(96.9% reduction)	106<br>(92.6% reduction)	195<br>(86.4% reduction)
Note: the original library had 1439 fungal extracts.			

Table S2: Hit rates for random sampling of positive mode fungal data. To prove our method's increased hit rates are not an artifact of a reduced absolute library size, we created 1000 iterations of random sampling that were the same size as our rationally reduced library (**Table S1**). Hit rates against each activity assay were then calculated. The lower quartiles, medians, and upper quartiles for all assay hit rates are listed. In every case, our rational method (**Table 1**) outperforms the random sampling.

Activity assay	Lower quartile, median, and upper quartile hit rates for 50 random extracts (1000 iterations)	Lower quartile, median, and upper quartile hit rates for 116 random extracts (1000 iterations)	Lower quartile, median, and upper quartile hit rates for 216 random extracts (1000 iterations)
P. falciparum	8.00%<br>12.00%<br>14.00%	9.48%<br>11.21%<br>12.93%	9.72%<br>11.11%<br>12.50%
T. vaginalis	4.00%<br>8.00%<br>10.00%	6.02%<br>7.76%<br>9.04%	6.48%<br>7.87%<br>8.80%
Neuraminidase	0.00%<br>2.00%<br>2.00%	0.86%<br>1.72%<br>2.58%	1.39%<br>1.85%<br>2.31%

Table S3: Summary of rational library sizes using publicly available data. Data was obtained from reference [22], as deposited in the MassIVE repository under accession number MSV000087728. This collection consisted of a broad taxonomy of pre-fractionated plant extracts.

	Number of Extracts to 80% Maximum Scaffold diversity	Number of Extracts to 95% Maximum Scaffold diversity	Number of Extracts to 100% Maximum Scaffold diversity
Public Data (1,600 total)	104 (93.5% reduction)	233 (85.4% reduction)	408 (74.5% reduction)

Table S4: Higher hit rates for rational libraries, for negative mode analysis of fungal samples. We assessed whether building scaffolds and rational libraries based on the negative polarity MS/MS data yielded the same results as analysis of data collected in positive mode. We found similar patterns, with an increased hit rate in the rational libraries for each activity assay.

Activity assay	Hit rate in full Library	Hit rate in the 80% scaffold diversity library	Hit rate in the 95% scaffold diversity library	Hit rate in the 100% scaffold diversity library
P. falciparum	11.26%	26.83%	18.75%	13.22%
T. vaginalis	7.64%	12.20%	12.50%	9.77%
Neuraminidase	2.57%	7.32%	4.17%	3.45%

Table S5: Classical molecular networking parameters. Parameters used for the positive polarity fungal data, public data, and negative polarity fungal data ("Analysis Parameters"). For low-resolution mimicking parameters, only positive polarity fungal samples were analyzed. See Data Availability section for GNPS job links.

	Analysis Parameters	Low-Resolution Mimicking Parameters
**Basic Options**		
Precursor Ion Mass Tolerance	0.02 Da	2 Da
Fragment Ion Mass Tolerance	0.02 Da	0.95 Da
**Advanced Network Options**		
Minimum Pairs Cosine	0.7	0.7
Network TopK	7	7
Maximum Connected Component Size	70	70
Minimum Matched Fragment Ions	4	4
Minimum Cluster Size	4	4
Maximum shift	500 Da	500 Da
**Advanced Filtering Options**		
Filter below Standard Deviation	0	0
Filter Precursor Window	filter	filter
Filter peaks in 50 Da Window	filter	filter
Filter Spectra from G6 as Blanks Before Networking	don't filter	don't filter
Minimum Peak Intensity	0	0
Filter Library	filter library	filter library

Table S6: Higher hit rates for rational libraries, using low-resolution mimicking parameters and positive mode analysis of fungal samples. To the same positive polarity fungal extract data, classical molecular networking was performed with parameters recommended for low-resolution LC-MS/MS data (See **Table S5** for parameters). The scaffolds and rational libraries were built using this output. We found similar patterns between the low-resolution mimicking and regular parameters, evident by an increased hit rate in the rational libraries for each activity assay.

Activity assay	Hit rate in full Library	Hit rate in the 80% scaffold diversity library	Hit rate in the 95% scaffold diversity library	Hit rate in the 100% scaffold diversity library
P. falciparum	11.26%	13.64%	12.26%	12.31%
T. vaginalis	7.64%	15.91%	10.38%	9.23%
Neuraminidase	2.57%	4.55%	3.77%	4.62%

Table S7: Q-Exactive plus parameters used for fungal data collection. Summary of parameters used in the Thermo Scientific XCalibur software for the positive and negative fungal extract data collection.

Parameter	Value (positive polarity)	Value (negative polarity)
Default Charge State	1	1
Polarity	Positive	Negative
Runtime	12.5 min	12.5 min
**Tune data**		
Ion source	HESI	HESI
Capillary temp (°C)	320	320
Spray voltage (|V|)	3800	3000
Sheath gas (Thermo Arbitrary units)	35	35
Max spray current (μA)	100	100
Probe heater temp (°C)	350	350
S-lens Radiofrequency level (Thermo Arbitrary units)	50	50
Auxiliary gas (Thermo Arbitrary units)	10	10
Sweep gas (Thermo Arbitrary units)	0	0
**Full MS**		
Scan Range	100-1500 m/z	100-1500 m/z
Maximum Injection Time	246 ms	246 ms
Resolution (at m/z 200, full width at half maximum (FWHM))	70,000	70,000
AGC Target	1e6	1e6
**dd-MS2**		
Isolation Window	1.0 m/z	1.0 m/z
Maximum Injection Time	54 ms	54 ms
(N)CE/stepped(N)CE	20 ,40, 60	20, 40, 60
Resolution (at m/z 200, full width at half maximum (FWHM))	17,500	17,500
AGC Target	2e5	2e5

TopN	5	5
**dd Settings**		
Intensity threshold	1.5e5	1.5e5
Minimum AGC target	8.00e3	8.00e3
Dynamic exclusion	10 s	10 s
Exclude isotopes	on	on
Peptide match	preferred	preferred
AGC:Automatic Gain Control, N(CE): Normalized Collision Energy, HESI: Heated electrospray ionization		

Table S8: MZmine 2.53 parameters used for feature identification: Summary of parameters used on the MS data files in MZmine 2.53. Positive and negative ionization data were analyzed separately.

MZmine 2.53 parameters		
	Positive Ionization	Negative Ionization
**Feature Detection**		
MS1 Retention Time (min)	0.2-12.5	0.2-12.5
MS1 Noise level	5E5	5E5
MS2 Retention Time (min)	0.2-12.5	0.2-12.5
MS2 Noise level	1E3	1E3
**Chromatogram Builder**		
Minimum Group Size	5	5
Group Intensity Threshold	5E5	5E5
Minimum Highest Intensity	1E6	1E6
m/z tolerance (ppm)	10	10
**Chromatogram Deconvolution**		
Algorithm	Local Minimum Search	Local Minimum Search
Chromatogram Threshold	20%	20%
Search Minimum (min)	0.15min	0.15 min
Minimum Relative height	25%	26%
Minimum Absolute height	1E6	1E6
Minimum ratio	1.15	1.3
Peak Duration (min)	0.05-1 min	0.05-1 min
m/z range for MS2	0.01 Da	0.01Da
RT range for MS2 (min)	0.2	0.2
**Deisotoping**		
m/z tolerance (ppm)	10	10
RT tolerance (min)	0.2	0.2
Monotonic shape	yes	yes

Maximum Charge	3	3
Representative isotope	Most intense	Most intense
**Join Aligner**		
m/z tolerance (ppm)	10 ppm	10 ppm
Weight for m/z	1	1
RT tolerance (min)	0.2 min	0.2 min
Weight for RT	1	1
**Filtering**		
Keep peaks with MS2 scan	yes	yes
Reset peak number ID	yes	yes

Supplemental Figures

Fig. S1: Number of features significantly correlated to bioactivity from each assay, along with known molecule derivatives identified in the analysis. In our bioactivity correlation analysis, we found no overlap between significant features or scaffolds between activity assays. As a proof-of-concept, within each assay, we were able to identify MS2 fragmentation matches to derivatives of small molecules with known antimicrobial activity, leucinostatin and altersetin.

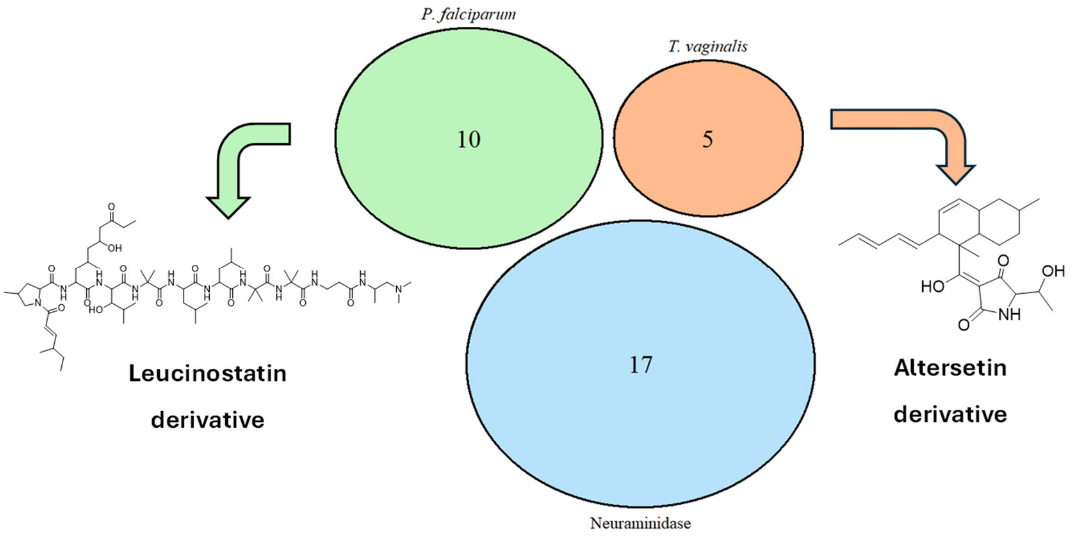

Fig. S2: Annotation overlap between positive and negative ionization modes.
Summary of similar annotations found by GNPS molecular networking software.

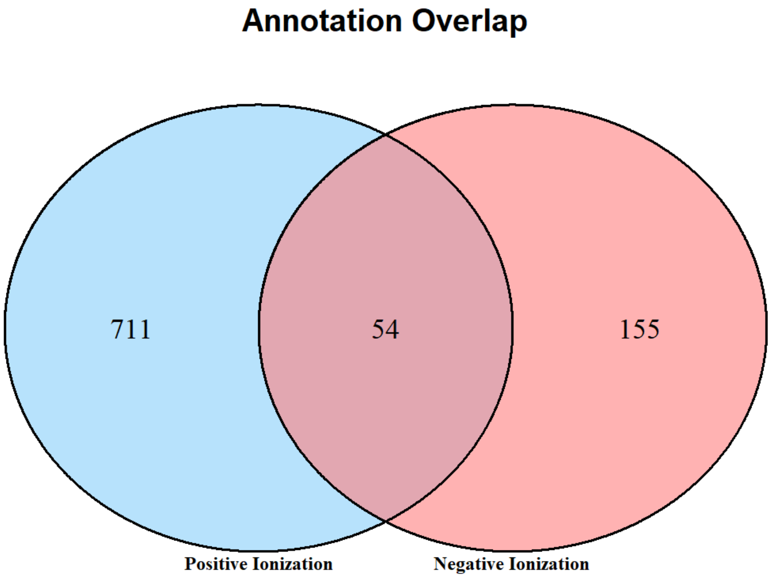

Fig. S3: Rapid accumulation of scaffold diversity with our rational library building method, for negative mode analysis of fungal samples. Scaffold accumulation for random sample iterations (50 iterations, each until 100% scaffold diversity reached) was outperformed by our rational library selection method.

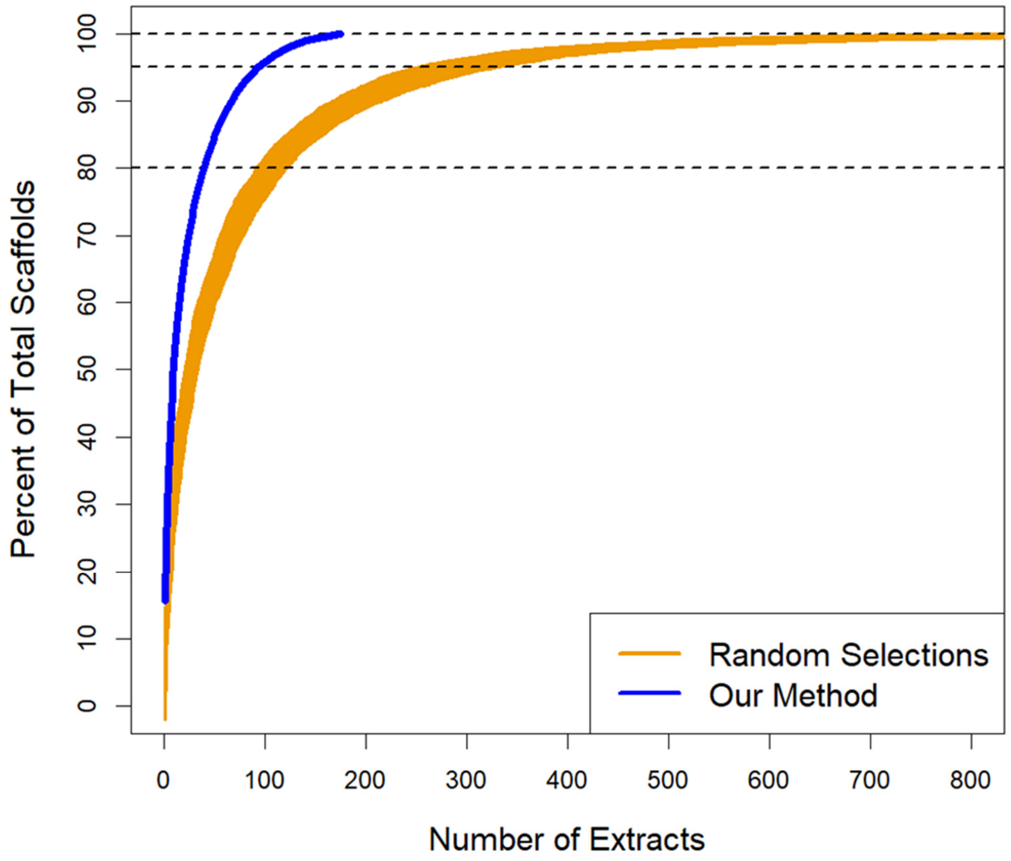

Fig. S4: Rapid accumulation of scaffold diversity with our rational library building method, using low-resolution-mimicking parameters and positive mode analysis of fungal samples. Scaffold accumulation of random sample iterations (50 iterations, each until 100% scaffold diversity reached), compared to our rational library selection method applied on data processed with low resolution-mimicking parameters.

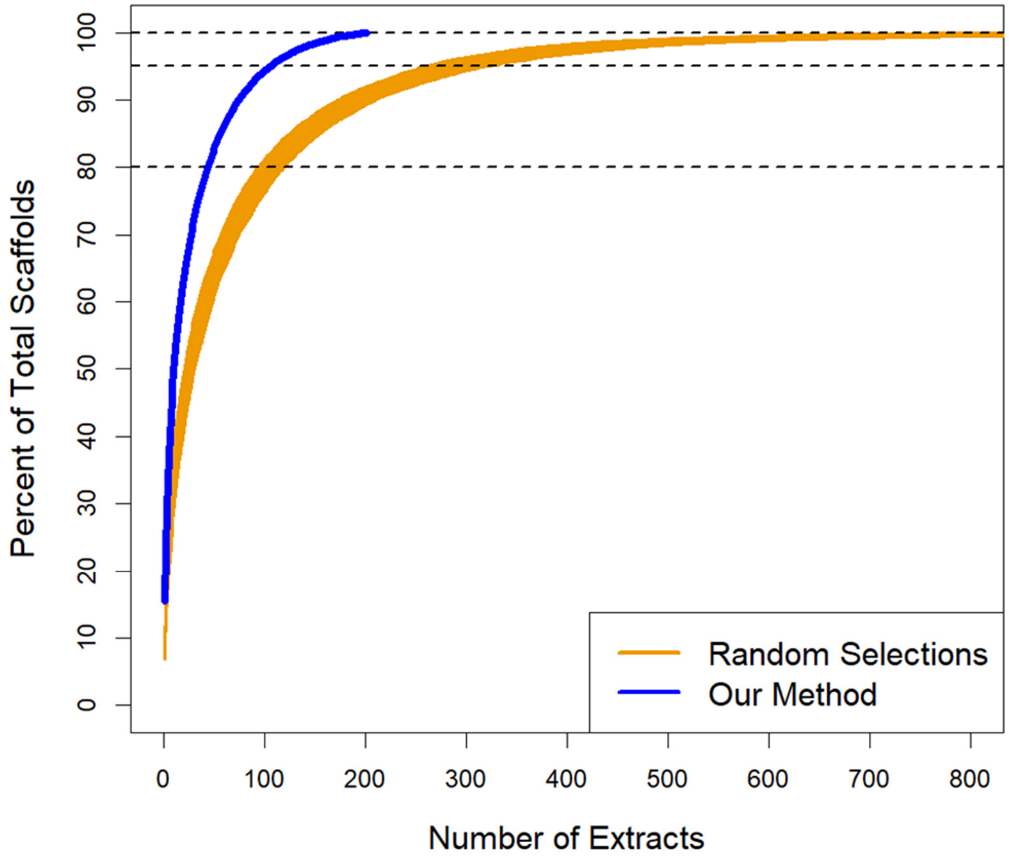

Fig. S5: Comparison of fungal genera presence in bioactive samples versus the full library. Data points above the y = x line indicate genera that are overrepresented in the active extracts relative to their abundance in the full library. Red markers denote statistically significant differences as determined by chi-squared analysis. Only genera with 10 or more extracts in the full library were included in this analysis.

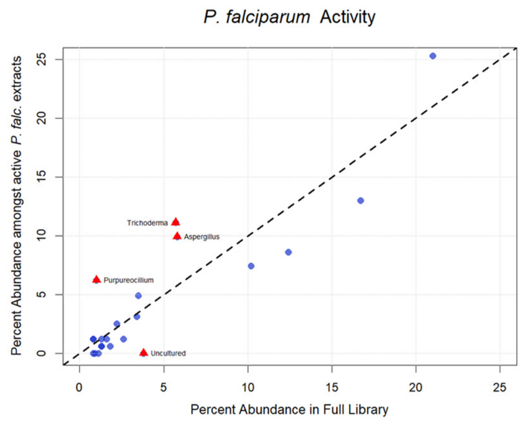

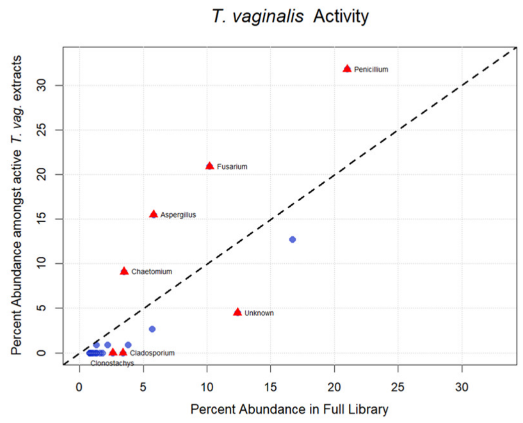

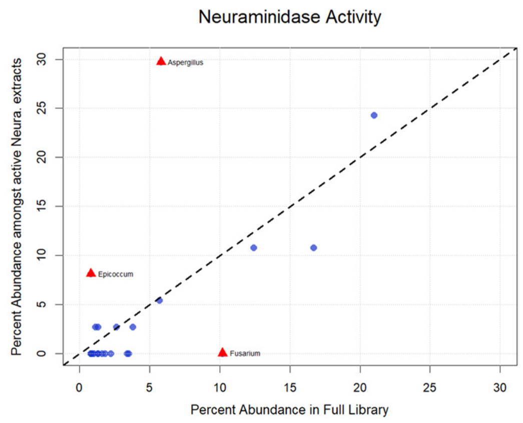

Fig. S6: Venn diagram showing active extracts and their overlap between assays. This shows active crude extracts between the 3 activity assays performed on the full library. Notably, there is minimal overlap between active extracts, other than 50 extracts with both *P. falciparum* and *T. vaginalis* activity, indicating the presence of molecules in these extracts with broad anti-parasitic properties.

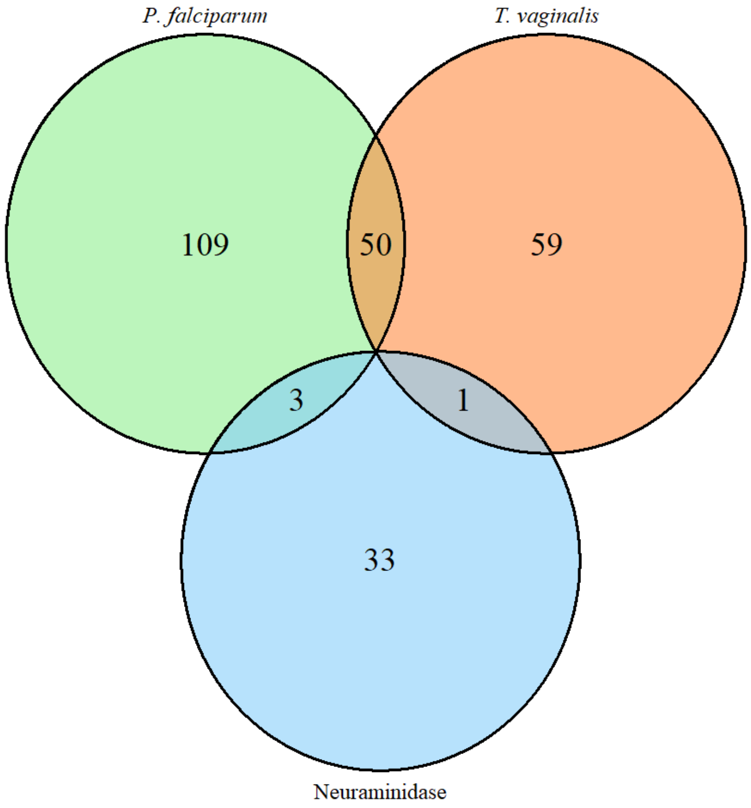

Fig. S7: **Fungal samples collection sites.** Summary of the origins of the fungal extracts used for analysis.

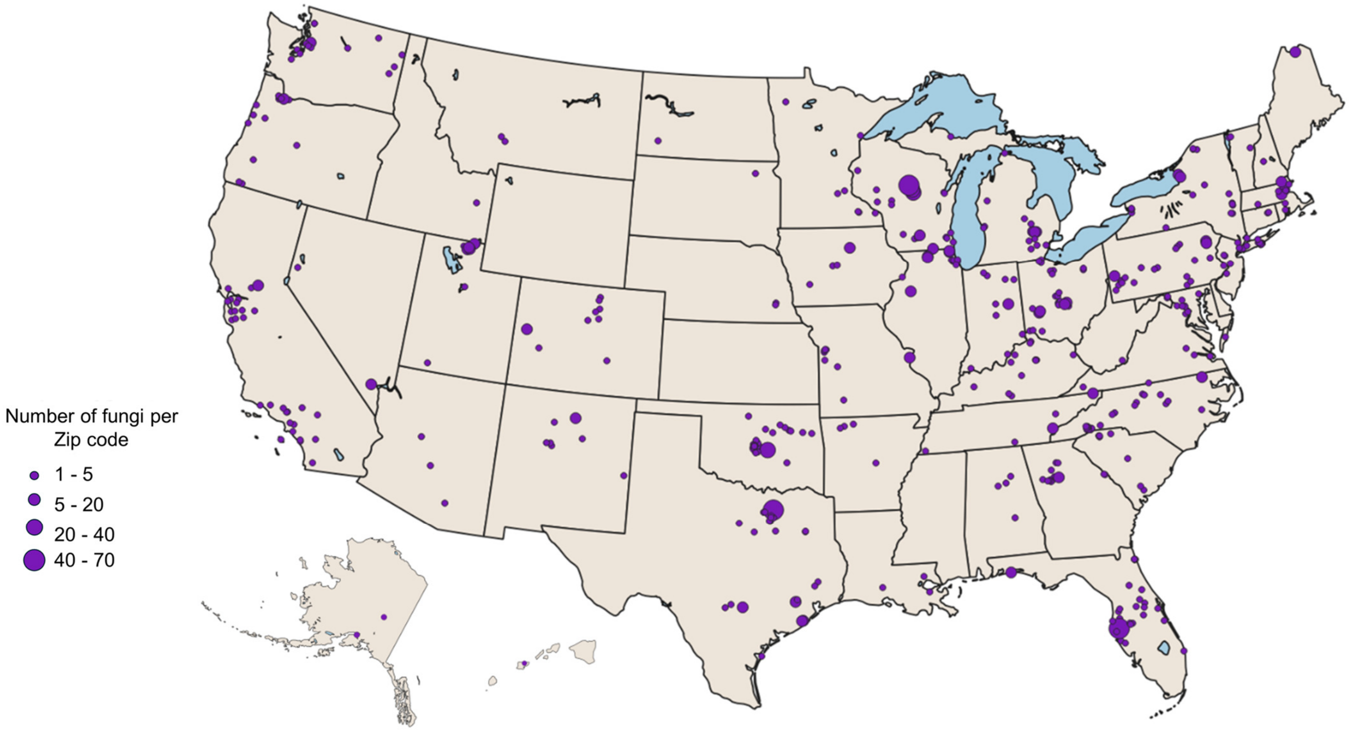

Fig. S8: Fungal samples collection sites, for active extracts. Summary of the origins of the active fungal extracts identified for each of the 3 assays. Green dots represent anti-*P. falciparum* activity, orange represents anti-*T. vaginalis* activity, and blue dots represent anti-neuraminidase activity. The size of the dots correlates to the number of active isolates from that area.

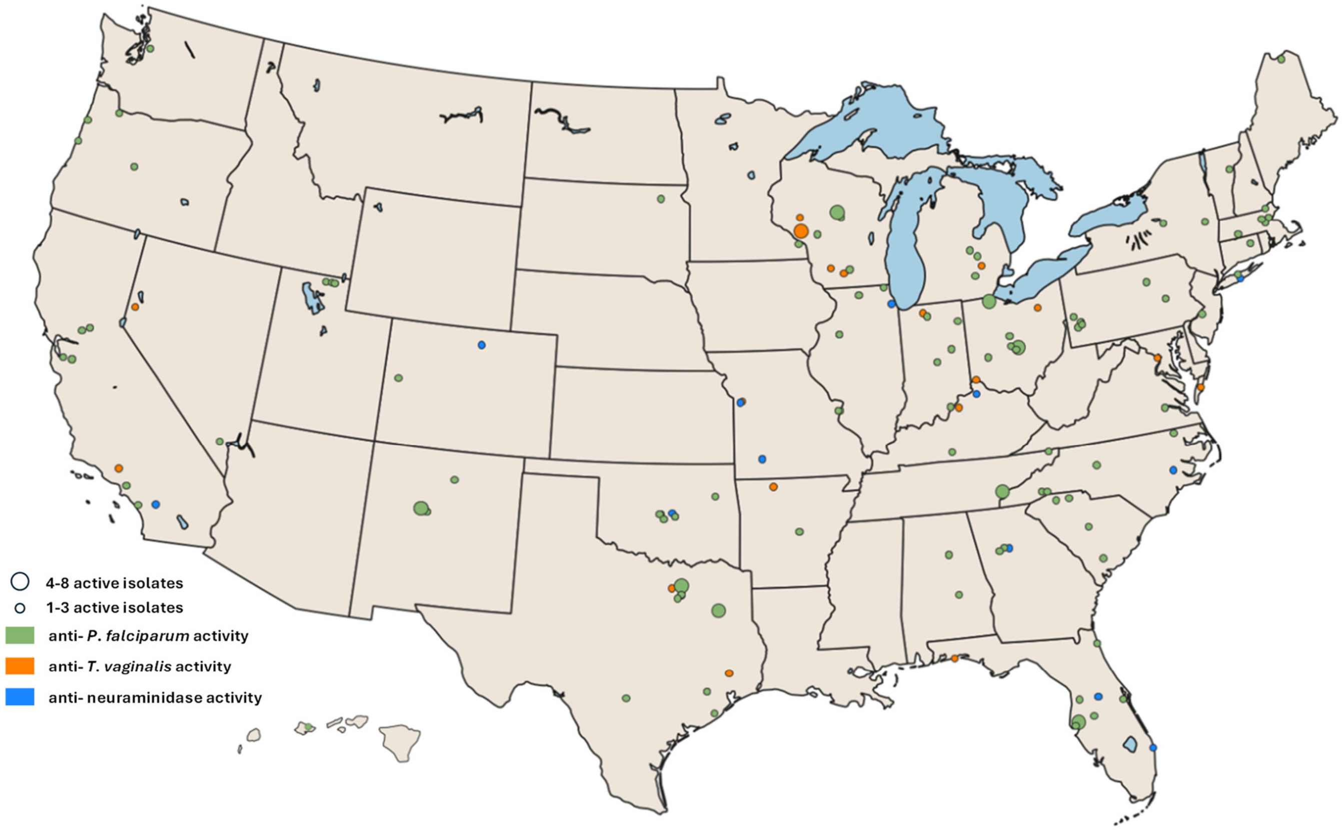

Fig. S9: Instructions for node table download from the classical molecular networking job page. When the classical molecular networking job is complete, access to a completed job status page appears (A). From there, select “Direct Cytoscape Preview/Download”, which leads to (B). Download the file and open Cytoscape. You will need to have the Cytoscape software installed. The networking job should appear and look similar to (C). Select “Export table to file” and save the file in a desired location as a .csv file.

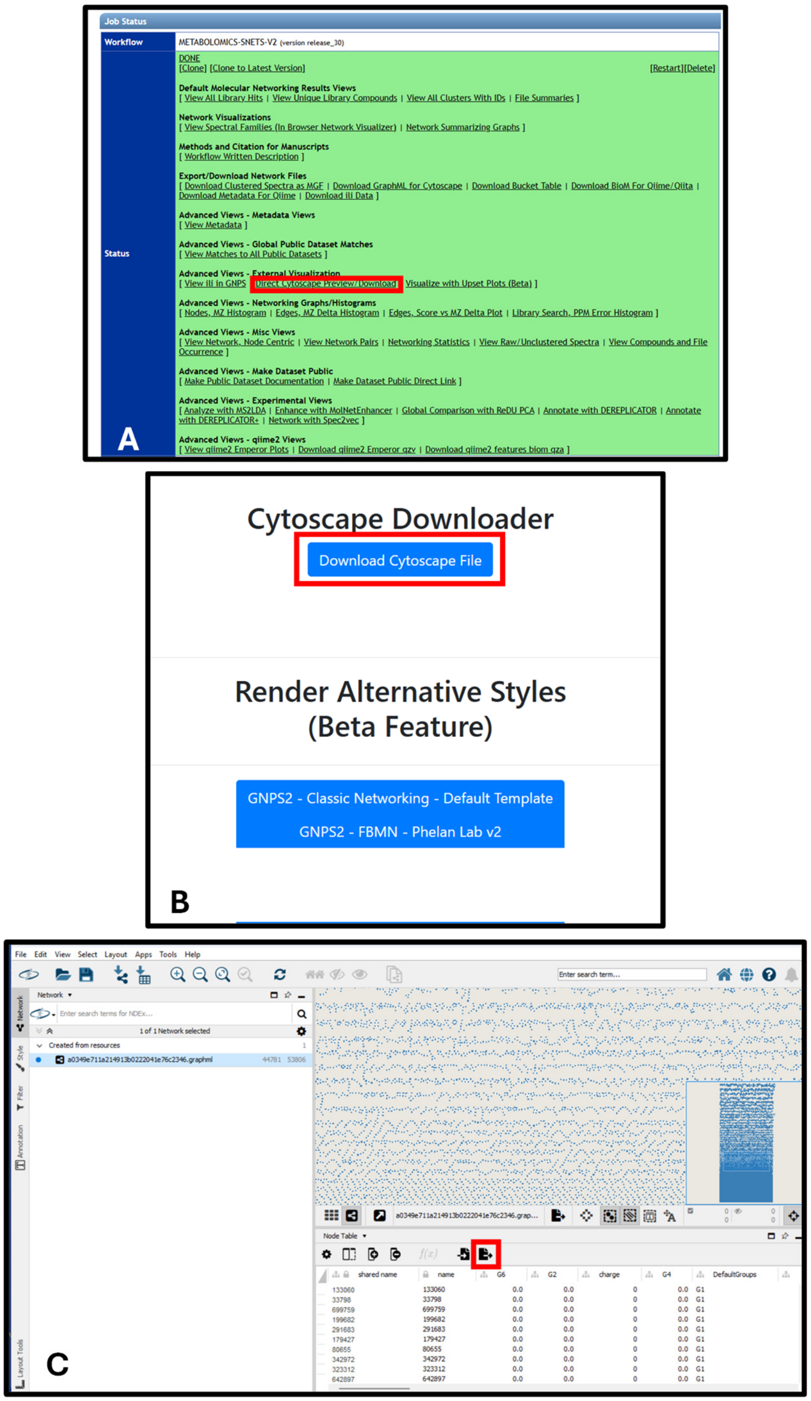

Supplementary Data (excel workbook):

Supplemental Data Sheet 1: Significant bioactive features for each ionization method. Counts of features significantly correlated with bioactivity, categorized by ionization method (positive or negative ion mode). On the left, the table outlines the method used for rational library generation. Rational libraries are created using Classical Molecular Networking (CMN) scaffolds, representing jobs for positive, negative, or low-resolution-mimicking positive ionization. For each CMN job, results from the three bioassays are presented. Within the table, counts of total significant features, and their retention in the rational libraries is provided. Since the same data was used for all correlation calculations, the total number of significant features in the full dataset remains constant for each bioassay, irrespective of the CMN job.

Supplemental Data Sheet 2: Changing classical molecular networking parameters has little impact on library size and hit rates. Summary of hit rates and library sizes with different multiple molecular networking parameters. Note that other than the parameters listed in the table, all other parameters are the same as listed in Table S5.

Supplemental Data Sheet 3: Genera origins of full and rational libraries: Summary of each genera count/percentages in the full libraries, and the count/percentages in the rational libraries. A Chi-squared significance test p-value is also listed to readily identify genera significantly over or underrepresented in the rational libraries

Supplemental Data Sheet 4: Fungal metadata. Summary of each fungal extract, the zip code the sample was collected, the soil extract origin, and the genus.
